# Elective interval thoracic endovascular aortic repair versus medical management for uncomplicated type B aortic dissection among Medicare patients

**DOI:** 10.1016/j.xjse.2026.100101

**Published:** 2026-02-06

**Authors:** Waseem Lutfi, Yu Zhao, Nicholas J. Goel, Madison A. Grasty, Alexandra E. Sperry, Michael A. Catalano, Chase R. Brown, Kendall M. Lawrence, Wilson Y. Szeto, Grace J. Wang, Nimesh D. Desai

**Affiliations:** a Division of Cardiovascular Surgery, University of Pennsylvania, Philadelphia, Pa; b Department of Biostatistics, Epidemiology, and Informatics, University of Pennsylvania, Philadelphia, Pa; c Division of Vascular Surgery, University of Pennsylvania, Philadelphia, Pa

**Keywords:** type B aortic dissection, thoracic endovascular aortic repair, interval TEVAR, medical management, Medicare

## Abstract

**Objective:**

To compare elective interval thoracic endovascular aortic repair (TEVAR) with ongoing medical management (MM) within 90 days for uncomplicated type B aortic dissection (uTBAD).

**Methods:**

Medicare MedPar files from 2001 to 2020 were queried for patients with acute uTBAD who were discharged after index hospitalization without operative intervention. The exposure was elective interval TEVAR versus MM within the first 90 days. Outcomes were overall survival, aortic reintervention, and reintervention-free survival. Propensity score matching, Kaplan-Meier analysis and Fine-Gray regression landmarking at 90 days were used.

**Results:**

In total, 23,697 patients were discharged without surgical intervention after admission for acute uTBAD. Within 90 days from diagnosis, 22,857 patients underwent MM, 399 elective TEVAR, and 441 emergent/urgent TEVAR. Matching between MM and elective TEVAR resulted in a well-balanced nearly 27:1 match with 10,608 patients in the MM cohort and 399 in the elective TEVAR cohort. The mean age was 73.5 years, and 51% of patients were female. 90-day mortality was 10.4% for MM and 4.3% for elective TEVAR. Landmarking at 90 days, there was no associated difference in overall survival with 5-year mortality of ∼63%. Similarly, there was no associated difference in reintervention-free survival. The 5-year cumulative incidence of aortic reintervention for MM was 11% and for TEVAR was 14% (*P* = .04).

**Conclusions:**

Among Medicare patients with acute uTBAD, there was no associated difference in overall survival or reintervention free survival between ongoing MM and elective interval TEVAR within 90 days.

## Graphical Abstract

**Figure:**
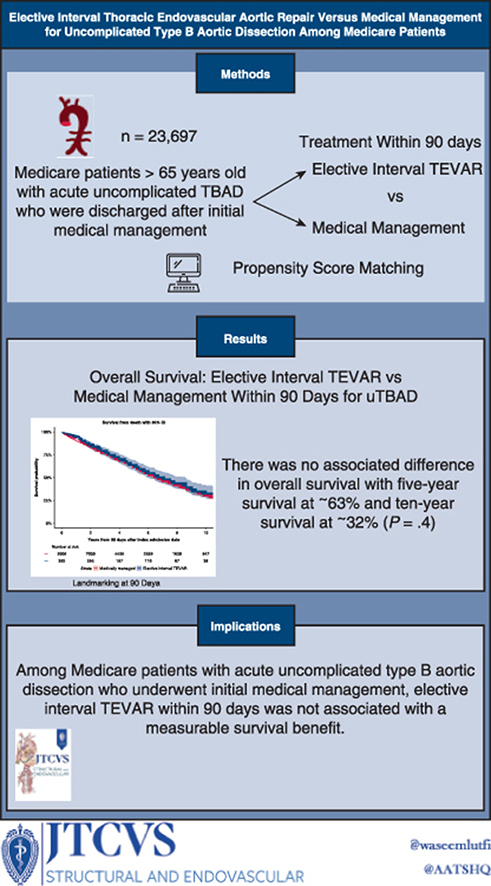

**Figure:**
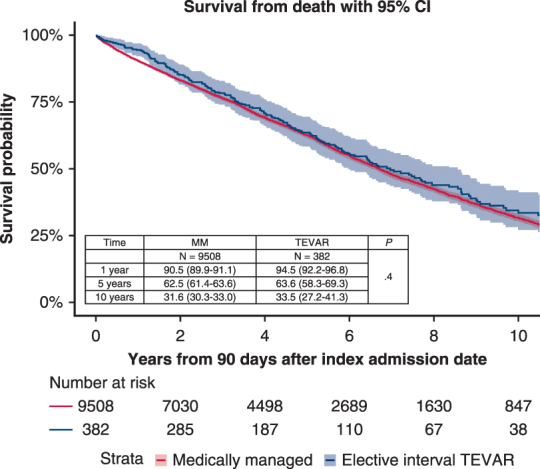
Overall survival: elective interval TEVAR vs medical management within 90 days for uTBAD.

**Central Message**

Among Medicare patients with acute uncomplicated type B aortic dissection who underwent initial medical management, elective interval TEVAR was not associated with a measurable survival benefit.

**Perspective**

In patients with a mean age of 73.5 with acute uncomplicated type B aortic dissection, elective interval TEVAR showed no associated difference in overall survival or aortic reintervention-free survival compared with ongoing medical management alone. Although arguably safe in this older age cohort, the utility of TEVAR may be limited.

Current guidelines recommend ongoing medical management (MM) and close clinical follow-up for all patients with acute uncomplicated type B aortic dissection (uTBAD).^1^^,^^2^ Meanwhile, thoracic endovascular aortic repair (TEVAR) has been recommended as a reasonable intervention in patients with high-risk features and suitable anatomy to reduce the risk of long-term aorta-related complications and promote aortic-remodeling. Early clinical trials have shown promising results. Investigators from the randomized Investigation of Stent Grafts in Aortic Dissection (INSTEAD-XL) trial found similar all-cause mortality at 2 years and an all-cause mortality benefit landmarking at 2 years for TEVAR compared with MM.^3^ The Acute Dissection Stent Grafting or Best Medical Treatment (ADSORB) trial demonstrated that TEVAR was superior to MM at 1 year with regards to false-lumen thrombosis, aortic dilatation, and rupture.^4^ Several larger nonrandomized cohort studies and their meta-analyses support these findings^5^, ^6^, ^7^, ^8^, ^9^; however, larger randomized trials are needed to confirm potential long-term benefit as well as the subgroups of patients most likely to benefit from endovascular intervention. Nevertheless, TEVAR has increased in use for patients with uTBAD nationally, with close to 9% of patients receiving an interval TEVAR within 1 year of acute TBAD.^10^

Currently, there is an international effort to determine the optimal treatment paradigm for uTBAD, with 3 multicenter trials recruiting and randomizing patients to early interval TEVAR versus MM for acute uTBAD with and without high-risk features: in the United States, the IMPRoving Outcomes in Vascular DisEase – Aortic Dissection (IMPROVE-AD) trial is expected to be completed in 2030,^11^ in Scandinavia the Scandinavian trial of Uncomplicated Aortic Dissection Therapy (SUNDAY) trial to be completed in 2026,^12^ and in the United Kingdom the Early aortic repair in patients needing endovascular/open surgery for type B aortic dissection (EARNEST) trial is expected to be completed in 2034.^13^ In the interim, additional studies are needed to help identify the role of TEVAR for uTBAD. There is also the additional consideration of when the optimal timing is to perform TEVAR for uTBAD, with conflicting reports in the literature regarding complications and mortality for TEVAR performed in the hyperacute window (<24 hours), acute window (1-14 days), subacute window (15-90 days), and chronic window (>90 days).^7^^,^^14^, ^15^, ^16^, ^17^, ^18^ With these questions in mind, this study sought to compare patients who underwent elective interval TEVAR within 90 days with patients who received ongoing MM alone for uTBAD using Medicare data.

## Methods

### Study Population and Data Source

**Figure 1:**
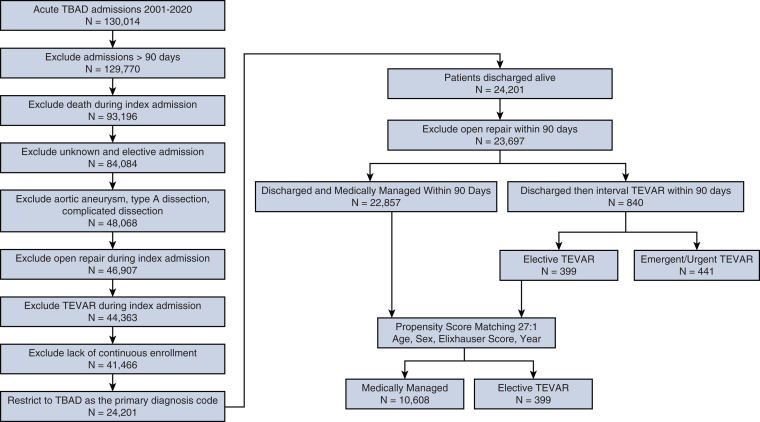
Patient selection. *TBAD*, Type B aortic dissection; *TEVAR*, thoracic endovascular aortic repair.

Inpatient Medicare MedPar files from 2001 to 2020 were queried for all patients with a first-time admission for the diagnosis of acute uTBAD who were discharged alive without receiving operative intervention (open aortic surgery or TEVAR) during that admission. The day of uTBAD diagnosis was defined as the date of admission. *International Classification of Diseases* (ICD) versions 9 and 10 Clinical Modification (CM) diagnosis codes as well as Procedure Coding System codes were used to identify patients with acute uTBAD using the coding schema previously published by Weissler and colleagues^5^ To summarize this schema, patients were required to have at least 1 year of Medicare enrollment and a primary diagnosis code for aortic dissection (ICD-9-CM: 441.01 and 441.03, ICD-10-CM: I71.01 and I71.03). Then, several exclusion criteria were applied, excluding patients with aortic rupture, organ malperfusion, stroke, and paralysis on admission, a previous hospitalization for aortic dissection, type A dissection, and patients who died during index admission. In addition to the selection criteria by Weissler and colleagues,^5^ we included only patients with an urgent, emergent, or trauma admission code and excluded patients with an elective or unknown admission code. We also excluded patients without continuous enrollment over the study period, those with their initial hospitalization lasting longer than 90 days, those who underwent TEVAR or open repair during their index admission, and those who underwent open repair within 90 days of uTBAD diagnosis (Figure 1). To summarize, we selected all patients from inpatient MedPar files from 2001 to 2020 with a first-time admission for uTBAD who were discharged alive without undergoing surgical intervention, and we excluded patients who underwent open surgical repair within 90 days of diagnosis. For clarity, in this study an “interval” TEVAR is a TEVAR that occurred after discharge from the index admission for uTBAD. Our primary comparison cohorts were patients who underwent elective interval TEVAR versus ongoing MM alone within 90 days of diagnosis.

This study was exempt from institutional review board review because it uses de-identified claims data per the University of Pennsylvania institutional review board policy. Informed consent was not required.

### Study Design and Outcomes

A retrospective cohort study design was performed with the exposure defined within the first 90 days from the day of uTBAD diagnosis: readmission for elective interval TEVAR within 90 days versus MM alone within 90 days. An elective admission code was required at the time of TEVAR, and patients who had emergent or urgent admission codes at the time of TEVAR were excluded from analysis (Figure 1). The primary outcome was overall survival landmarking at 90 days. Secondary outcomes were reintervention-free survival and the cumulative incidence of aortic reintervention with death as a competing risk landmarking at 90 days. Aortic reintervention was defined as any open or endovascular aortic procedure after 90 days for both the MM and TEVAR cohorts. Landmarking was used to negate immortality time bias because patients undergoing elective TEVAR could potentially have an immortal time up to 90 days during the exposure defining 90-day window. Covariates used in propensity score generation were age, sex, the Elixhauser comorbidity index,^19^ and the year of initial TBAD admission in 5-year periods: 2001-2005, 2006-2010, 2011-2015, and 2016-2020. Of note, TEVAR was not commercially available in the United States before 2005 and not approved for TBAD until 2013.

### Statistical Analysis

Baseline cohort characteristics were compared using Student *t* tests for continuous variables and χ^2^ tests for categorical variables. Propensity scores were generated using multivariable logistic regression modeling conditional on age, sex, Elixhauser comorbidity index, and 5-year time periods. N to one (N:1) propensity score matching was used to match for all previously mentioned covariates between exposure cohorts. Propensity scores were generated using the MatchIt R package (version 4.5.5),^20^ and N:1 matching was performed using greedy nearest-neighbor matching without replacement with a caliper width of 0.2, matching the MM cohort onto the smaller elective interval TEVAR cohort; caliper widths of 0.2 are common practice in epidemiologic research studies.^21^^,^^22^ The matching algorithm was repeated to reach the maximum number of matches that met balance criteria resulting in nearly a 27:1 match. Love plots demonstrating standardized mean differences before and after matching were used to assess matching balance of covariates with a 10% standardized mean difference cutoff for covariate match acceptability. Kaplan-Meier curves and log-rank tests were used to compare overall survival and reintervention-free survival out to 10 years between exposure cohorts.

Due to death as a competing risk, aortic reintervention was compared using Fine-Gray subdistribution hazard models and displayed using a cumulative incidence function. Subhazard ratios with 95% CIs were used for exposure point estimates.

To assess for any potential residual unmeasured confounding after propensity score matching, we used the 2 falsification end points of urinary tract infection (UTI) and hip fractures comparing the cumulative incidences of each on Fine-Gray analysis with death as a competing risk. These endpoints have been used in previous studies.^5^

Lastly, because ICD coding was used to generate diagnoses and procedures by querying available ICD codes from inpatient claims, any missing data not captured are unknown. Thus, we did not have any missing data to analyze, and there was no role for imputation.

All tests were 2 sided and an alpha of 5% was used as the cutoff for statistical inference. Statistical software used included R Statistical Software (version 4.2.1; R Core Team 2023).

### Additional Subgroup Analyses

Using the indicator of hospital admission source available among MedPar files, we described the admission source among the excluded subgroup of patients undergoing urgent or emergent TEVAR within 90 days of diagnosis. We also compared their overall survival with patients undergoing elective interval TEVAR within 90 days from the time of surgery. Lastly, we investigated the admission sources for interval TEVAR after 90 days among patients who initially underwent MM.

### Sensitivity Analyses

We conducted 5 sensitivity analyses to bolster our primary analysis. First, to define our exposure in this study, we chose the 90-day TEVAR exposure window in line with the current Society for Vascular Surgery/Society of Thoracic Surgeons reporting standards for TBAD^23^ that define >90 days from the time of TBAD diagnosis as the chronic window. To understand the impact of this choice on our outcome, we repeated our primary analysis using a cutoff of 180 days to define our exposure window. Thus, we compared elective interval TEVAR within 180 days versus patients undergoing MM alone within 180 days. Similarly, we used N:1 propensity score matching to balance covariates and performed a survival analysis landmarking at 180 days.

Next, the comparator cohort in our primary analysis for elective interval TEVAR within 90 days was all patients who underwent initial MM for 90 days. Given the absence of anatomical data available in this administrative dataset, it is impossible to know which patients in the MM cohort did not have indications for TEVAR or were unsuitable candidates. However, one can surmise that patients who underwent initial MM then had an elective interval TEVAR after 90 days of diagnosis had similar indications for TEVAR and were not precluded for unmeasured anatomical variables. Thus, we conducted a sensitivity analysis comparing all patients who underwent interval TEVAR within 90 days with patients who underwent interval TEVAR within 91 days to 1 year. Due to immortality time bias in patients who had an interval TEVAR after 90 days, we measured survival from the time of TEVAR in both cohorts. Our 2 comparison cohorts were the 399 patients who underwent interval TEVAR within 90 days to 394 patients who underwent interval TEVAR between 91 days to 1 year. We used unadjusted Kaplan-Meier curves to compare survival as well as cox-regression adjusted for all the covariates in our primary matched analysis.

In our third sensitivity analysis, rather than defining a fixed exposure window for elective interval TEVAR and using propensity score methods, we conducted 2 separate Cox proportional hazards models with elective interval TEVAR as a time-dependent exposure variable with survival measured from the time of index admission to 90 days, and another model from the time of index admission to 180 days. These Cox models were adjusted for all the covariates in our primary matched analysis.

In our fourth sensitivity analysis, we stratified our analysis into 2 time periods, before and after Food and Drug Administration approval for TEVAR in TBAD in 2013 with 8 years of follow-up. In our final sensitivity analysis, we compared overall survival between patients treated with TEVAR within 30 days and those treated between 31 and 90 days. The impetus for this final analysis is the inability with the Medicare database to identify patients who may have developed high risk features or converted to a complicated TBAD if not coded as such, as those treated earlier may be a proxy for those findings.

## Results

**Figure E1:**
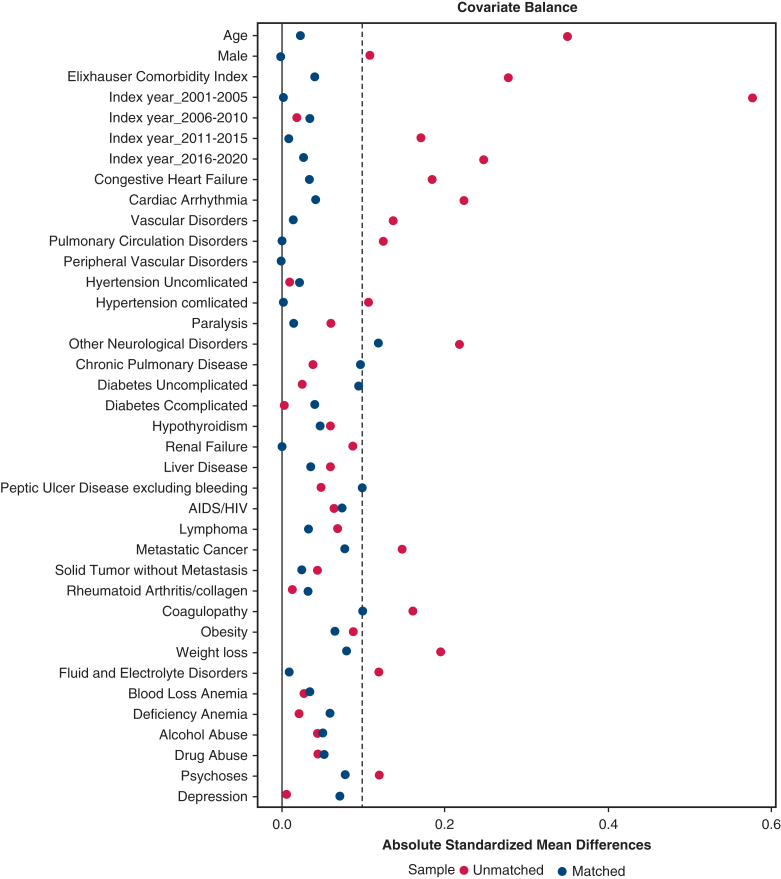
Love plots of covariate balance before and after propensity score matching.

After patient selection, 22,857 (96.5%) patients underwent MM alone after index admission for uTBAD, whereas 399 (1.7%) underwent elective interval TEVAR within 90 days; 441 (1.8%) patients underwent urgent or emergent interval TEVAR within 90 days, and these patients were excluded from the primary analysis. The mean age was 76.5 years, 43.8% of patients were male, and 78.3% were White, 15.1% were Black, and 6.6% were another racial category. Table 1 shows the cohort characteristics by exposure cohort before and after 27:1 propensity score matching conditional on age, sex, Elixhauser comorbidity index, and year. Notably, the mean age of the matched cohort was 73.5 years. In addition, Table 1 includes the 31 individual components of the Elixhauser comorbidity index, noting a successful match that also balanced nearly all 31 index components within a 10% standardized mean difference. Figure E1 shows the Love plots for the propensity score matching.

**Table 1:** Cohort characteristics before and after propensity score matching

N	Before matching			After matching		
	MM	TEVAR	SMD	MM	TEVAR	SMD
	22,857	399		10,608	399	
Matched covariates						
Age, y, mean (SD)	76.6 (10.3)	73.3 (8.3)	0.35	73.6 (9.9)	73.3 (8.3)	0.026
Male, n (%)	9990 (43.7)	196 (49.1)	0.109	5209 (49.1)	196 (49.1)	<0.001
Elixhauser index, mean (SD)	4.82 (2.35)	4.23 (1.92)	0.278	4.1 (1.9)	4.2 (1.9)	0.048
Five-year periods			0.597			0.038
2001-2005, n (%)	5141 (22.5)	15 (3.8)		413 (3.9)	15 (3.8)	
2006-2010, n (%)	5223 (22.9)	88 (22.1)		2498 (23.5)	88 (22.1)	
2011-2015, n (%)	5098 (22.3)	119 (29.8)		3124 (29.4)	119 (29.8)	
2016-2020, n (%)	7395 (32.4)	177 (44.4)		4574 (43.1)	177 (44.4)	
Individual Elixhauser Comorbidity Index components, n (%)						
Congestive heart failure	5145 (22.5)	61 (15.3)	0.185	1476 (13.9)	61 (15.3)	0.039
Arrhythmia	8929 (39.1)	114 (28.6)	0.223	3246 (30.6)	114 (28.6)	0.044
Valvular disease	4953 (21.7)	65 (16.3)	0.137	1664 (15.7)	65 (16.3)	0.016
Pulmonary circulation disorders	1486 (6.5)	15 (3.8)	0.125	401 (3.8)	15 (3.8)	0.001
Peripheral vascular disease	22,857 (100.0)	399 (100.0)	<0.001	10,608 (100.0)	399 (100.0)	<0.001
Hypertension, uncontrolled	17,079 (74.7)	300 (75.2)	0.011	7872 (74.2)	300 (75.2)	0.023
Hypertension, controlled	5235 (22.9)	74 (18.5)	0.108	1980 (18.7)	74 (18.5)	0.003
Paralysis	236 (1.0)	2 (0.5)	0.061	68 (0.6)	2 (0.5)	0.018
Other neurologic disorders	1984 (8.7)	14 (3.5)	0.217	674 (6.4)	14 (3.5)	0.132
COPD	7167 (31.4)	118 (29.6)	0.039	2659 (25.1)	118 (29.6)	0.101
Diabetes, uncontrolled	3173 (13.9)	59 (14.8)	0.026	1210 (11.4)	59 (14.8)	0.100
Diabetes, controlled	1168 (5.1)	20 (5.0)	0.004	435 (4.1)	20 (5.0)	0.044
Hypothyroidism	3889 (17.0)	59 (14.8)	0.061	1383 (13.0)	59 (14.8)	0.050
Renal failure	3744 (16.4)	53 (13.3)	0.087	1399 (13.2)	53 (13.3)	0.003
Liver disease	676 (3.0)	8 (2.0)	0.061	273 (2.6)	8 (2.0)	0.038
Peptic ulcer disease	310 (1.4)	8 (2.0)	0.050	77 (0.7)	8 (2.0)	0.111
HIV/AIDS	50 (0.2)	0 (0.0)	0.066	26 (0.2)	0 (0.0)	0.070
Lymphoma	169 (0.7)	1 (0.3)	0.070	53 (0.5)	1 (0.3)	0.040
Metastatic cancer	384 (1.7)	1 (0.3)	0.147	109 (1.0)	1 (0.3)	0.097
Solid tumor without metastasis	1001 (4.4)	14 (3.5)	0.045	322 (3.0)	14 (3.5)	0.026
Rheumatoid arthritis/collagen	1334 (5.8)	22 (5.5)	0.014	503 (4.7)	22 (5.5)	0.035
Coagulopathy	2240 (9.8)	22 (5.5)	0.162	866 (8.2)	22 (5.5)	0.105
Obesity	2183 (9.6)	49 (12.3)	0.088	1083 (10.2)	49 (12.3)	0.066
Weight loss	1494 (6.5)	10 (2.5)	0.195	442 (4.2)	10 (2.5)	0.092
Fluid and electrolyte disorders	7320 (32.0)	106 (26.6)	0.120	2762 (26.0)	106 (26.6)	0.012
Blood loss anemia	438 (1.9)	6 (1.5)	0.032	113 (1.1)	6 (1.5)	0.039
Deficiency anemia	897 (3.9)	14 (3.5)	0.022	252 (2.4)	14 (3.5)	0.067
Alcohol use disorder	623 (2.7)	8 (2.0)	0.047	295 (2.8)	8 (2.0)	0.051
Drug use disorder	623 (2.7)	8 (2.0)	0.047	300 (2.8)	8 (2.0)	0.054
Psychosis	407 (1.8)	2 (0.5)	0.121	142 (1.3)	2 (0.5)	0.088
Depression	3040 (13.3)	54 (13.5)	0.007	1175 (11.1)	54 (13.5)	0.075

*MM*, Medical management; *TEVAR*, thoracic endovascular aortic repair; *SMD*, standardized mean difference; *SD*, standard deviation; *COPD*, chronic obstructive pulmonary disease.

### Events Within 90 Days of Diagnosis Before Landmarking and After Matching

Rates of mortality within 90 days from uTBAD diagnosis were 10.7% among patients undergoing ongoing MM and 4.3% among patients who had ad elective interval TEVAR. Five patients who underwent TEVAR (1.3%) had an aortic reintervention within 90 days from uTBAD diagnosis. The mean time to elective interval TEVAR after diagnosis was 45.8 days; 39 patients (9.7%) had TEVAR within 1 to 14 days and 360 patients (90.3%) had TEVAR within 15 to 90 days.

### Primary and Secondary Outcomes Landmarking at 90 Days

**Figure 2:**
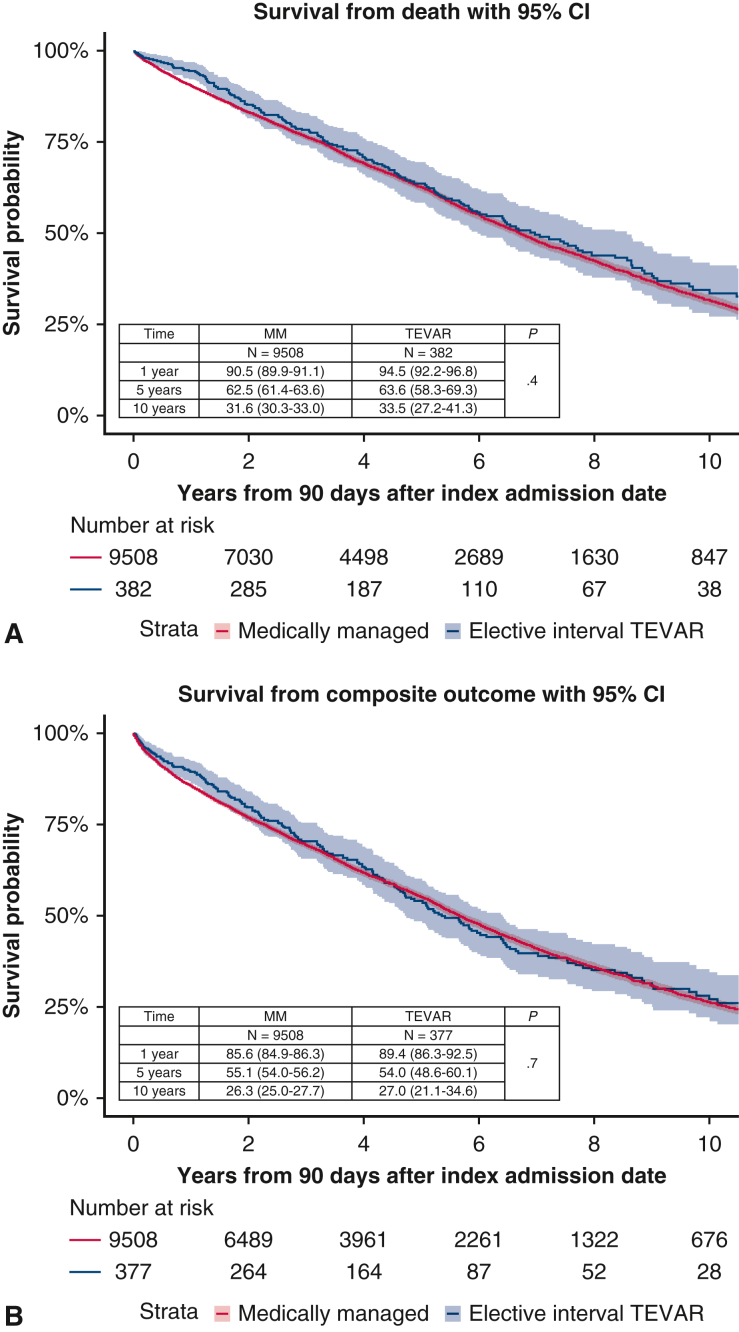
A, Overall survival among matched cohorts landmarking at 90 days with 95% CI. B, Reintervention-free survival among matched cohorts landmarking at 90 days with 95% CI. *TEVAR*, Thoracic endovascular aortic repair.

Figure 2, *A*, shows overall survival curves by exposure cohort landmarking at 90 days. There was no associated difference in overall survival with 5-year survival at ∼63% and 10-year survival at ∼32% (hazard ratio [HR], 0.93; 95% CI, 0.80-1.09, *P* = .4). Figure 2, *B*, shows reintervention-free survival and similarly shows no associated difference in reintervention-free survival out to 10 years (HR, 0.98; 95% CI, 0.86-1.13, *P* = .7).

**Figure 3:**
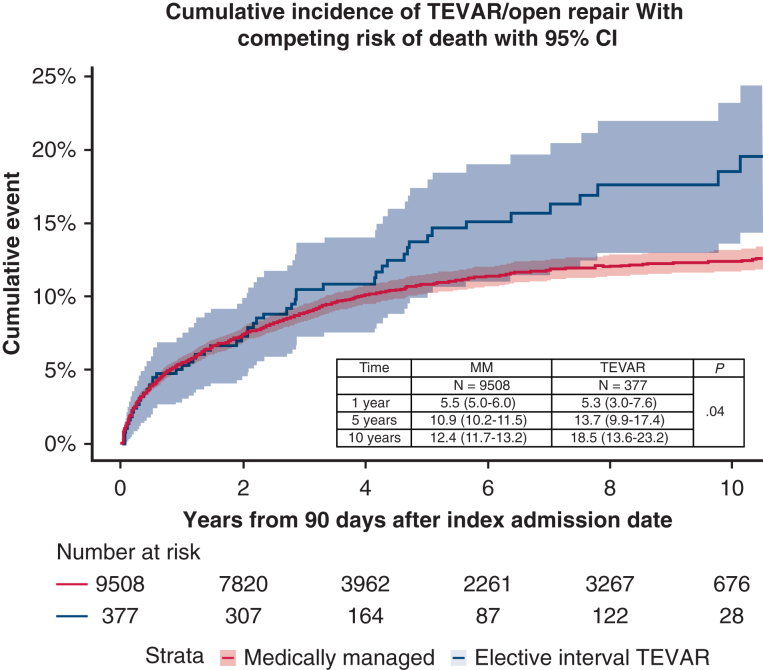
Cumulative incidence of aortic reintervention among matched cohorts landmarking at 90 days with 95% CI. *TEVAR*, Thoracic endovascular aortic repair.

We also analyzed the incidence of aortic reintervention as an independent outcome from survival. Figure 3 shows the cumulative incidence of aortic reintervention landmarking at 90 days derived from Fine-Gray regression with death as a competing risk. There was no associated difference in the cumulative incidence of aortic reintervention at 1 year between TEVAR (5.3%) versus ongoing MM (5.5%); however, by 5 years TEVAR had a greater cumulative incidence (14% vs 11%), which persisted out to 10 years (19% vs 12%), *P* = .042. Table E1 shows the subhazard ratios and 95% CIs. With regards to the type of aortic reintervention, for MM 34.8% were open and 65.2% were endovascular, whereas for TEVAR, 22.0% were open and 78.0% were endovascular (*P* = .052).

### Falsification End-Point Analysis

**Figure 4:**
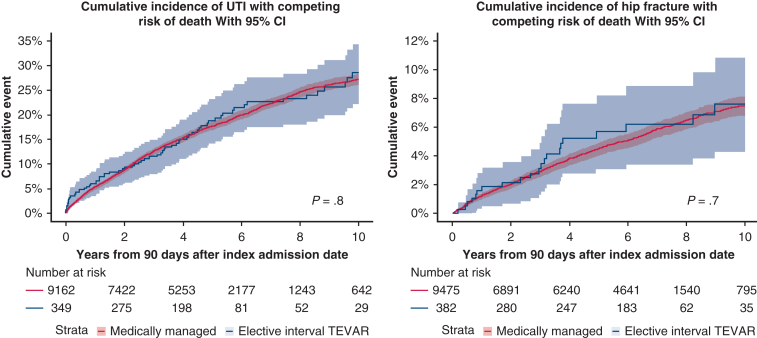
Falsification end-point analysis – cumulative incidences of UTI and hip fracture with 95% CI. *UTI*, Urinary tract infection.

To increase the robustness of our analysis and assess for unmeasured confounding after matching, the cumulative incidences of UTI and hip fracture were compared among matched cohorts landmarking at 90 days. As shown in Figure 4, there were no associated difference in the cumulative incidences of UTI (*P* = .8) or hip fracture (*P* = .7) between elective interval TEVAR or ongoing MM within 90 days.

### Additional Subgroup Analyses

**Figure 5:**
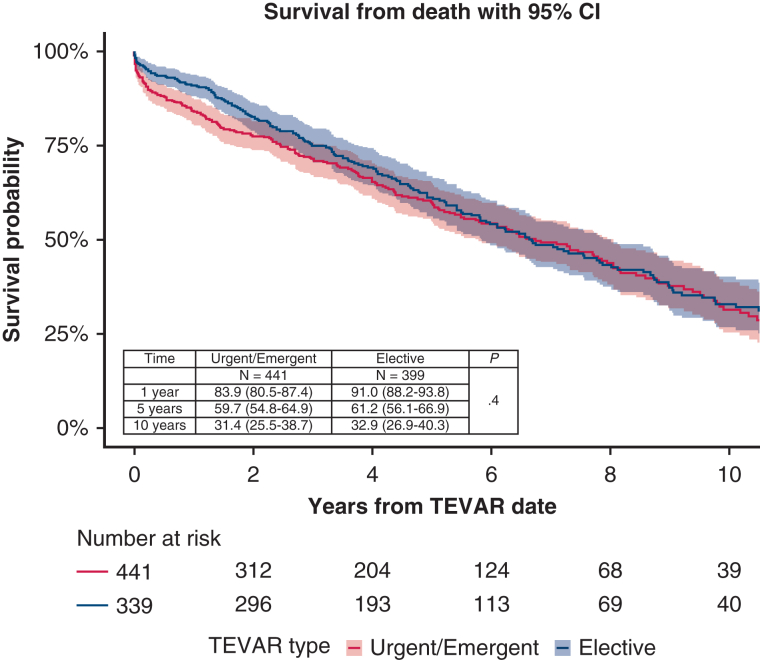
Overall survival - urgent/emergent interval TEVAR versus elective interval TEVAR within 90 days with 95% CI. *TEVAR*, Thoracic endovascular aortic repair.

In total, 441 patients had undergone an urgent/emergent admission for interval TEVAR within 90 days of diagnosis after initial successful MM and discharge. Table 2 shows the source of admission for patients who had an urgent/emergent TEVAR within 90 days. We found that 56.8% of patients were admitted via physician or clinic referral and 43.0% were admitted after transfer from an outside hospital or through the emergency department. Compared with patients who had an elective interval TEVAR within 90 days, patients who had an urgent/emergent interval TEVAR had greater initial mortality with worse associated survival at 1 year (83.9% vs 91.0%, *P* = .002) but no statistically significant difference in long-term overall survival (Figure 5, *P* = .4). Table E2 shows the cohort characteristics of urgent/emergent TEVAR versus elective interval TEVAR.

**Table 2:** Source of admission by TEVAR timing

Admission source	Urgent/emergent TEVAR within 90 d	Interval TEVAR after 90 d – MM cohort
N	441	1251
Physician referral	217 (49.1%)	807 (64.5%)
Clinic referral	34 (7.7%)	279 (22.3%)
Transfer from another hospital	157 (35.6%)	125 (10.0%)
Emergency department	32 (7.4%)	27 (2.2%)
Other	1 (0.2%)	13 (1.0%)

*TEVAR*, Thoracic endovascular aortic repair; *MM*, medical management.

Among the entire unmatched cohort of 22,857 patients who underwent MM within the first 90 days, 1251 patients (5.5%) had an interval TEVAR after 90 days; 71.8% (898) were performed during an elective admission, whereas 27.9% (349) were performed during an urgent/emergent admission. 86.8% of patients were admitted via physician or clinic referral and 12.2% were admitted after transfer from an outside hospital or through the emergency department (Table 2).

### Sensitivity Analyses

**Figure E2:**
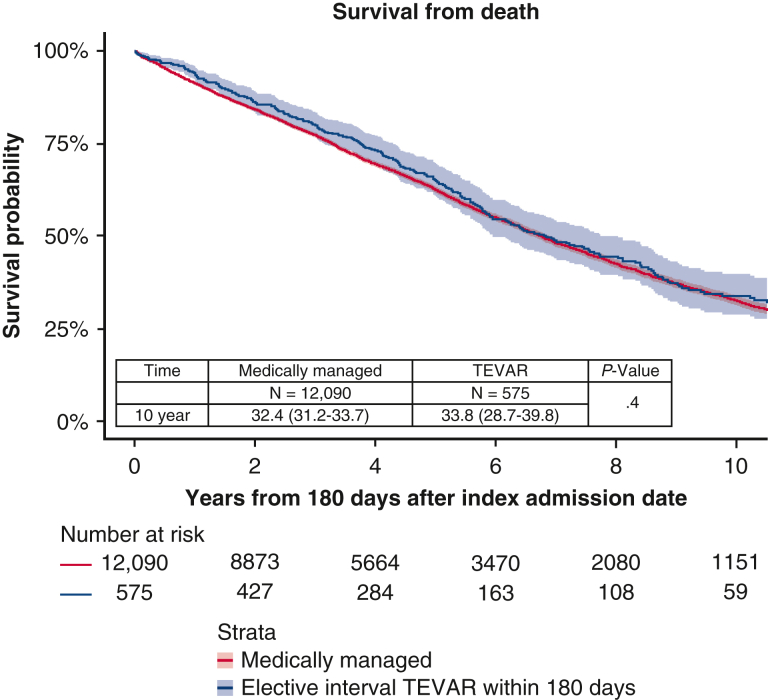
Overall survival between matched cohorts with a 180-day TEVAR cutoff landmarking at 180 days with 95% CI. *TEVAR*, Thoracic endovascular aortic repair; *MM*, medical management.

In our first sensitivity analysis, we repeated our primary analysis using a 180-day exposure window. Using N:1 matching, a well-balanced near 21:1 match was achieved: 12,090 patients who underwent MM alone within 180 days were matched to 575 patients undergoing elective interval TEVAR within 180 days. Figure E2 shows the Kaplan-Meier curves for overall survival between matched cohorts. Like our primary analysis, there was no associated difference in overall survival with 10-year survivals of 32.4% for MM versus 33.8% for TEVAR (*P* = .3).

**Figure E3:**
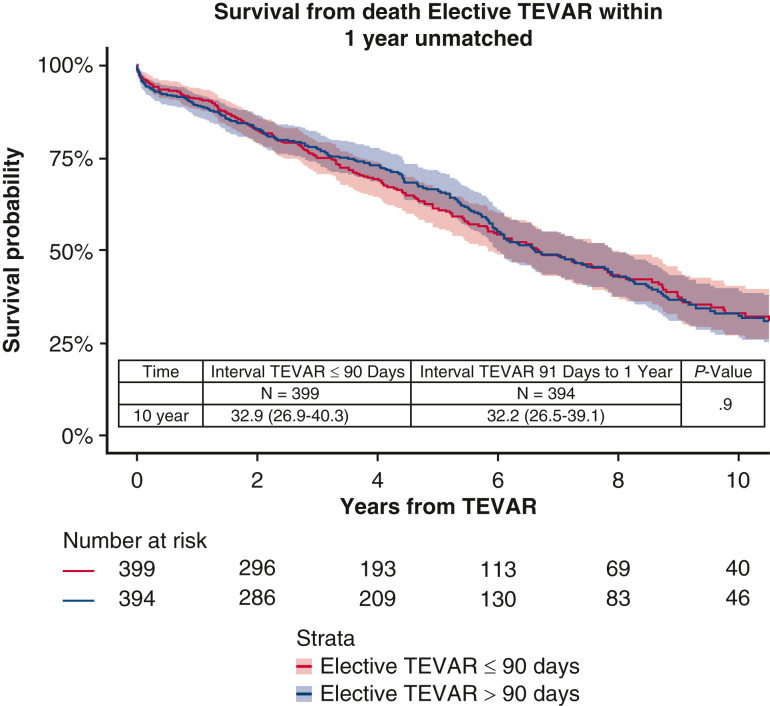
Unadjusted overall survival between elective interval TEVAR within 90 days versus elective interval TEVAR within 91 days to 1 year with 95% CI. *TEVAR*, Thoracic endovascular aortic repair.

We next compared overall survival between the 399 patients who underwent elective interval TEVAR within 90 days to the 394 patients who underwent elective interval TEVAR between 91 days to 1 year. Figure E3 shows the unadjusted Kaplan-Meier curves for overall survival between these two TEVAR cohorts and showed no associated difference in overall survival (*P* = .9). On multivariable Cox regression, compared to TEVAR within 90 days, TEVAR after 90 days was not associated with a difference in overall survival (HR, 0.93; 95% CI, 0.76-1.14, *P* = .473). Table E3 shows the multivariable Cox regression model.

Next, we conducted multivariable Cox models with elective interval TEVAR as a time-dependent exposure variable. Table E4 shows the Cox model with elective interval TEVAR exposure up to 90 days and Table E5 shows the Cox model with elective interval TEVAR exposure up to 180 days. Neither TEVAR within 90 days (HR, 0.96; 95% CI, 0.84-1.11, *P* = .612) or within 180 days (HR, 0.95; 95% CI, 0.85-1.06, *P* = .368) were associated with a significant difference in overall survival.

**Figure E4:**
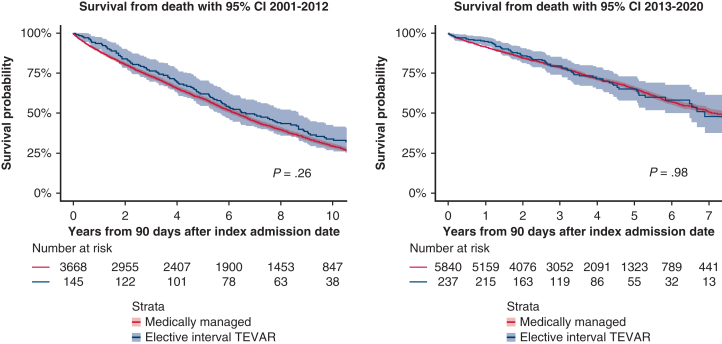
Overall survival among matched cohorts landmarking at 90 days before and after Food and Drug Administration approval in 2013 with 95% CI. *TEVAR*, Thoracic endovascular aortic repair.

**Figure E5:**
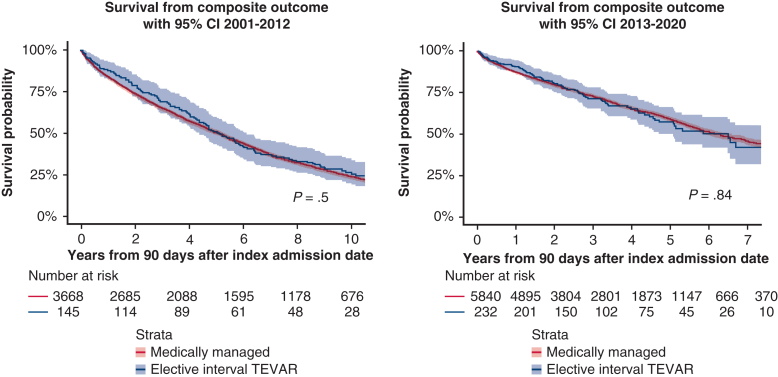
Reintervention-free survival among matched cohorts landmarking at 90 days before and after approval from the Food and Drug Administration in 2013 with 95% CI. *TEVAR*, Thoracic endovascular aortic repair.

**Figure E6:**
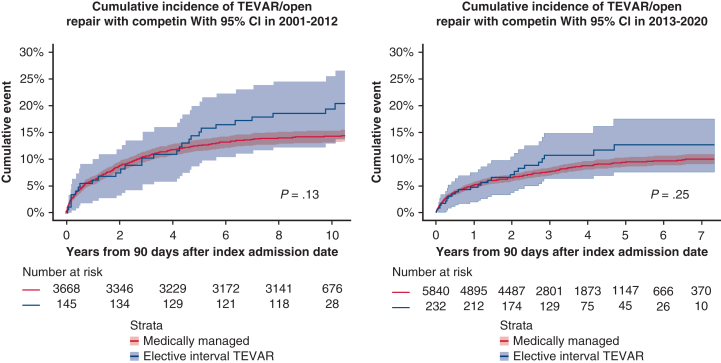
Cumulative incidence of aortic reintervention among matched cohorts landmarking at 90 days before and after Food and Drug Administration approval in 2013 with 95% CI. *TEVAR*, Thoracic endovascular aortic repair.

**Figure E7:**
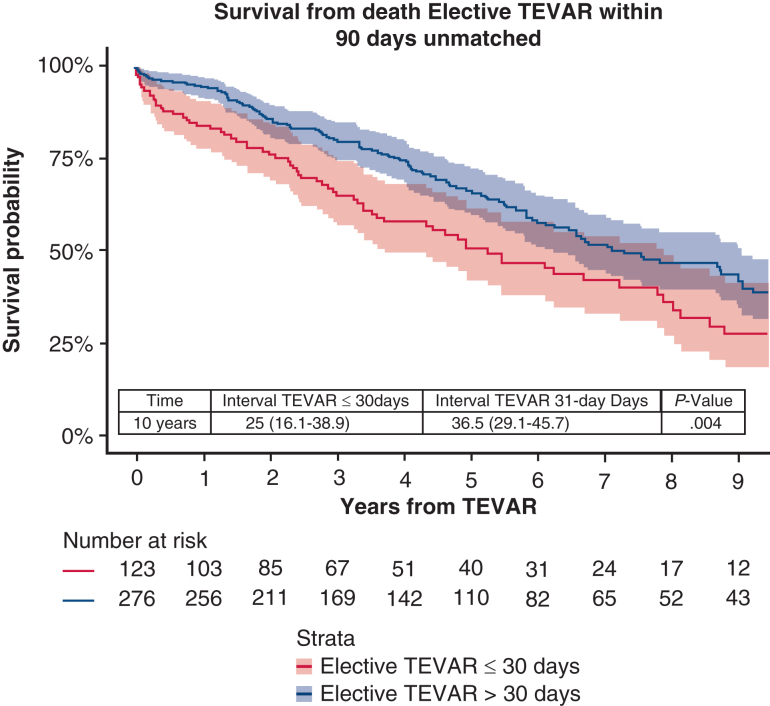
Unadjusted overall survival between elective interval TEVAR within 30 days versus elective interval TEVAR within 31 to 90 days with 95% CI. *TEVAR*, Thoracic endovascular aortic repair.

When stratifying our analysis into 2 time periods, before and after 2013 Food and Drug Administration approval of TEVAR for TBAD, we found similar results to our primary analysis in both time periods, as shown in Figures E4-E6. In our last sensitivity analysis comparing patients who had an interval TEVAR within 30 days with those who had an interval TEVAR within 31 to 90 days, patients who had a TEVAR within 30 days had worse overall survival out to 10 years (25.0% vs 36.5%, *P* = .004) as shown in Figure E7.

## Discussion

In this review of inpatient Medicare data from 2001 to 2020, we examined patients with a mean age of 73.5 years with acute uTBAD who had successful initial nonoperative management to discharge and then compared the outcomes of patients who had elective interval TEVAR to ongoing MM within the first 90 days. Notably, 96.5% of patients underwent MM within 90 days and only 1.7% had an elective interval TEVAR. In matched comparisons, we found no associated difference in overall survival with 10-year survival at ∼32%. Although the cumulative incidence of aortic reintervention was slightly greater in the TEVAR cohort, reintervention-free survival was similar. Lastly, even though all patients survived initial hospitalization to discharge, the 90-day mortality was >10% for the entire cohort with most early deaths in the MM cohort.

Our study follows a similar study design to a 2023 study by Weissler and colleagues,^5^ with a notable difference in our selection criteria. Weissler and colleagues^5^ used inpatient Medicare data to compare TEVAR versus MM within 30 days of admission for acute uTBAD. Like our cohort, the mean age was 75 years old. The authors found that 16% of patients with uTBAD underwent upfront TEVAR, which was not associated with any mortality difference from MM at 5 years but was associated with an increased risk of stroke and readmission. As their study provides insight into the impact of upfront TEVAR for acute uTBAD in older patients, our study aimed to analyze a separate question: the impact of elective interval TEVAR after an initial course of MM resulting in discharge. Nonetheless, we found similar findings with no survival benefit associated with elective interval TEVAR out to 10 years and increased aortic reintervention among patients who underwent TEVAR. Perhaps not surprising, given the older demographic of our cohort with a mean age of 73.5 years, as patients may not live long-enough to demonstrate any potential survival benefits of either treatment strategy. Another interesting difference in our studies is that Weissler and colleagues^5^ reported increased incidences of the falsification end points of hip fracture and UTI among their TEVAR cohort after propensity score inverse probability weighting, indicating potential residual confounding. However, our study found no associated difference in the incidences of hip fracture or UTI between cohorts. We believe our selection criteria requiring patients survive to discharge without surgical intervention, as well as excluding patients undergoing urgent/emergent TEVAR, likely removed a source of unmeasured confounding. Another potential contribution was our choice to use N:1 propensity score matching rather than inverse probability weighting (as used by Weissler and colleagues^5^) may have made our MM cohort more comparable with the TEVAR cohort for our estimation of an average treatment effect on the treated.

With limited randomized data available, multiple retrospective cohort studies have also compared TEVAR with MM for uTBAD. Their findings have been well summarized in several systematic-review articles and meta-analyses. A 2023 meta-analysis by Sa and colleagues^6^ pooled time-to-event data from 10 studies comparing TEVAR with MM for a total patient sample of 17,906 patients and found improved all-cause mortality and aortic-related mortality for TEVAR compared to MM. It should be noted, however, that most of the included studies except for the aforementioned study by Weissler and colleagues^5^ included cohorts with a mean age range of 48 years to 66 years, which were much lower than the mean age of our cohort. In addition, most patients received TEVAR for uTBAD in the acute window of 14 days as opposed to our study in the subacute window of 15 to 90 days. Another detail of importance is that 1 of the 10 studies, a randomized trial by Wei and colleagues^24^ that showed improved mortality with TEVAR over MM, was recently retracted because of methodologic flaws. Nonetheless, 2 other meta-analyses of similar published cohorts by Wang and colleagues^25^ and Hossack and colleagues^26^ found similar conclusions favoring long-term outcomes for TEVAR over MM. The findings among these younger age cohorts provide the basis for current ongoing and anticipated randomized trials.

Aside from the potential long-term benefit of TEVAR over MM for patients with uTBAD, another important question is the optimal timing of TEVAR to reduce postoperative complications in the setting of TBAD, in particular the acute versus subacute TBAD window. A lack of consistency in the nomenclature for TBAD timing let to early variations in reporting; however, the most recent Society for Vascular Surgery/Society of Thoracic Surgeons reporting standards for TBAD have provided standardized definitions for TBAD timing from time of symptoms onset: hyperacute <24 hours, acute 1 to 14 days, subacute 15 to 90 days, and chronic greater than 90 days.^23^ Several early studies demonstrated increased postoperative complications associated with TEVAR performed in the acute versus subacute window for TBAD,^14^^,^^16^^,^^27^ a finding noted in a review by Jubouri and colleagues^28^ However, a new meta-analysis of 20 studies and 792 patients now provides some conflicting evidence.^15^ Our analysis does not have enough patients undergoing elective interval TEVAR within the acute window to compare surgical outcomes between subacute and acute elective interval TEVAR timing. However, our study demonstrates that patients who underwent elective interval TEVAR in primarily the subacute window have similar mortality to patients undergoing MM. Thus, elective interval TEVAR among this older age cohort was comparatively safe and did not confer a greater long-term risk than patients undergoing conservative MM. This finding should provide some evidence that surgeons are selecting well older patients for elective interval TEVAR to avoid increased mortality. Nonetheless even with surgeon selection, our data show no measurable benefit up to 10 years after elective interval TEVAR among older patients. Consequently, surgeons should recognize that elective interval TEVAR has not been shown to provide survival benefit in patients who are approximately 73 years old with uTBAD. Furthermore, it could be argued that the 441 patients in our study who underwent an urgent/emergent interval TEVAR within 90 days could be considered failures of a MM first approach; however, despite initial worse associated survival, they ultimately had similar associated long-term survival to elective interval TEVAR and could be considered rescued. Thus, our data suggest that surgeons could justifiably refrain from offering early elective TEVAR intervention to older patients. Perhaps in very well-selected older patients with a substantially prolonged life expectancy, elective TEVAR still has a role to play in enhancing long-term outcomes. However, data from further observational studies and upcoming clinical trials are still needed to elucidate any potential survival benefit associated with elective interval TEVAR.

### Limitations

Our study was an attempt to understand the risks and benefits of elective interval TEVAR after a patient has been discharged after successful MM for a new diagnosis of uTBAD. First, as a retrospective observational study, our study can only draw associations rather than causation or noninferiority between our exposure cohorts and measured outcomes. Furthermore, our study may simply be underpowered to detect clinically meaningful differences between comparison groups, particularly given the small TEVAR cohort of 399 patients. A null finding in an observational study using claims data should not be interpreted as evidence of clinical equivalence.

Perhaps the biggest limitation to our study design is our inability to understand the initial management strategy at the time of discharge from index admission to allow for an intention to treat analysis. Surely there were patients whose initial plan was to undergo MM alone and close follow-up who then underwent elective or urgent TEVAR or had an early unexpected mortality. Similarly, patients planned for elective interval TEVAR in a subacute window may have progressed to complicated disease necessitating urgent TEVAR or early death. There are also the added elements of patient preference for intervention as well as changes in surgical plan based on unmeasured time-varying factors like patient symptoms, new imaging findings, and high-risk features all of which were unavailable. The high rate of 90-day mortality in the MM cohort likely reflects this early management dilemma and perhaps reflects patients that may have been unsuitable for surgical intervention. Further, our sensitivity analysis showing that patients who had an elective interval TEVAR within 30 days had worse overall survival compared to patients who had an elective interval TEVAR within 31 to 90 days supports the hypothesis that patients treated earlier were greater risk and “semi-urgent.” Landmarking at 90 days was our best attempt to isolate comparable patient risk profiles and negate immortality time bias. One could argue that with the high early mortality and our findings of no notable difference in survival or reintervention between the 2 cohorts, perhaps surgeons are doing a good job observing those that can be observed and selecting those to be intervened on. Furthermore, there was no associated difference in long-term overall survival between patients undergoing elective interval TEVAR versus urgent/emergent TEVAR within 90 days which is reassuring that delayed surgical management and progression to urgent/emergent intervention does not compromise long-term survival. A major limitation to our study is the lack of variable granularity and anatomic data necessary to assess the candidacy of patients for surgical treatment. We also have no laboratory value data. Ultimately these limitations cannot be overcome with this database and may be a source of unmeasured confounding. It is at least reassuring that our falsification endpoint analysis does not demonstrate substantial unmeasured confounding between our comparison cohorts. With regards to patients undergoing aortic reintervention, a database limitation is the inability to distinguish between planned staged reinterventions and revisions for failed procedures. Another database limitation is ICD coding misclassification, which is unavoidable and would tend to bias our outcomes toward the null, an important consideration in our study with negative findings. Lastly, outcomes for older Medicare patients may not generalize to younger patients, and our findings and conclusions are thus restricted to the older demographic in this study.

## Conclusions

Among Medicare patients with acute uTBAD who underwent successful initial MM and discharge, comparing elective interval TEVAR with ongoing MM within 90 days, we found high rates of 90-day mortality at >10%, no associated difference in overall survival out to 10 years, a greater incidence of reintervention among patients who undergo TEVAR, but no associated difference in reintervention-free survival. In this older age cohort, our analysis does not demonstrate a measurable benefit to elective interval TEVAR over ongoing MM alone.

## Conflict of Interest Statement

Dr Desai has consulted for W. L. Gore & Associates and Terumo Aortic, which had no influence on the study design or conclusions. Dr Szeto has consulted for Abbott, Artivion, Edwards Lifesciences, Medtronic, and Terumo Aortic, for which he is an investigator, advisory board, and speaker. All other authors reported no conflicts of interest.

The *Journal* policy requires editors and reviewers to disclose conflicts of interest and to decline handling or reviewing manuscripts for which they may have a conflict of interest. The editors and reviewers of this article have no conflicts of interest.

## Figures and Tables

**Table E1:** Fine-Gray regression SHR

Admission source	SHR (95% CI)	*P* value
TEVAR/open repair	1.35 (1.03-1.75)	.028
Urinary tract infection	1.04 (0.81-1.31)	.767
Hip fracture	1.09 (0.71-1.68)	.705

*SHR*, Subhazards ratio; *TEVAR*, thoracic endovascular aortic repair.

**Table E2:** Cohort characteristics of elective interval TEVAR versus urgent/emergent interval TEVAR within 90 days

Admission source	Urgent/emergent	Elective	*P* value	SMD
N	441	399		
Age, y, mean (SD)	73.05 (9.52)	73.32 (8.31)	.667	0.03
Male	204 (46.3)	196 (49.1)	.447	0.057
Elixhauser index, mean (SD)	4.48 (2.13)	4.23 (1.92)	.075	0.124
Congestive heart failure	76 (17.2)	61 (15.3)	.504	0.053
Arrhythmia	137 (31.1)	114 (28.6)	.476	0.055
Valvular disease	56 (12.7)	65 (16.3)	.167	0.102
Pulmonary circulation disorders	30 (6.8)	15 (3.8)	.071	0.136
Peripheral vascular disease	441 (100.0)	399 (100.0)	NA	<0.001
Hypertension (uncontrolled)	344 (78.0)	300 (75.2)	.378	0.067
Hypertension (controlled)	84 (19.0)	74 (18.5)	.923	0.013
Paralysis	4 (0.9)	2 (0.5)	.774	0.049
Other neurologic disorders	32 (7.3)	14 (3.5)	.026	0.167
COPD	126 (28.6)	118 (29.6)	.808	0.022
Diabetes (uncontrolled)	65 (14.7)	59 (14.8)	1	0.001
Diabetes (controlled)	19 (4.3)	20 (5.0)	.749	0.033
Hypothyroidism	60 (13.6)	59 (14.8)	.696	0.034
Renal failure	54 (12.2)	53 (13.3)	.728	0.031
Liver disease	18 (4.1)	8 (2.0)	.125	0.121
Peptic ulcer disease	5 (1.1)	8 (2.0)	.458	0.07
HIV/AIDS	0	0	NA	<0.001
Lymphoma	3 (0.7)	1 (0.3)	.688	0.063
Metastatic cancer	4 (0.9)	1 (0.3)	.432	0.087
Solid tumor without metastasis	10 (2.3)	14 (3.5)	.384	0.074
Rheumatoid arthritis/collagen	38 (8.6)	22 (5.5)	.107	0.121
Coagulopathy	43 (9.8)	22 (5.5)	.03	0.16
Obesity	49 (11.1)	49 (12.3)	.675	0.036
Weight loss	21 (4.8)	10 (2.5)	.122	0.121
Fluid and electrolyte disorders	136 (30.8)	106 (26.6)	.197	0.095
Blood loss anemia	9 (2.0)	6 (1.5)	.744	0.041
Deficiency anemia	17 (3.9)	14 (3.5)	.934	0.018
Alcohol use disorder	12 (2.7)	8 (2.0)	.65	0.047
Drug use disorder	12 (2.7)	8 (2.0)	.65	0.047
Psychosis	6 (1.4)	2 (0.5)	.355	0.09
Depression	63 (14.3)	54 (13.5)	.83	0.022

*TEVAR*, Thoracic endovascular aortic repair; *SMD*, standardized mean difference; *SD*, standard deviation; *COPD*, chronic obstructive pulmonary disease.

**Table E3:** Cox proportional hazards regression for elective interval TEVAR within 90 days versus elective interval TEVAR within 91 days to 1 year

Admission source	HR (95% CI)	*P* value
TEVAR 91 d to 1 y	0.93 (0.76-1.14)	.473
Age, y	1.04 (1.02-1.05)	<.001
Male	1.21 (0.99-1.48)	.059
Elixhauser index	1.16 (1.1-1.22)	<.001
Index year		
2001-2005	Ref	Ref
2006-2010	0.95 (0.68-1.33)	.765
2011-2015	0.85 (0.6-1.21)	.369
2016-2020	0.76 (0.51-1.14)	.182

*TEVAR*, Thoracic endovascular aortic repair; *HR*, hazard ratio.

**Table E4:** Cox proportional hazards regression for elective interval TEVAR within 90 days as a time-dependent covariate

Admission source	HR (95% CI)	*P* value
TEVAR (time dependent)	0.96 (0.84-1.11)	.612
Age, y	1.05 (1.05-1.05)	<.001
Male	1.07 (1.04-1.1)	<.001
Elixhauser index	1.16 (1.16-1.17)	<.001
Index year		
2001-2005	Ref	Ref
2006-2010	0.93 (0.89-0.97)	<.001
2011-2015	0.83 (0.79-0.87)	<.001
2016-2020	0.69 (0.65-0.72)	<.001

*TEVAR*, Thoracic endovascular aortic repair; *HR*, hazard ratio.

**Table E5:** Cox proportional hazards regression for elective interval TEVAR within 180 days as a time-dependent covariate

Admission source	HR (95% CI)	*P* value
TEVAR (time dependent)	0.95 (0.85-1.06)	.368
Age, y	1.05 (1.05-1.05)	<.001
Male	1.07 (1.04-1.11)	<.001
Elixhauser index	1.16 (1.16-1.17)	<.001
Index year		
2001-2005	Ref	Ref
2006-2010	0.93 (0.89-0.97)	.001
2011-2015	0.83 (0.79-0.87)	<.001
2016-2020	0.69 (0.65-0.72)	<.001

*TEVAR*, Thoracic endovascular aortic repair; *HR*, hazard ratio.
